# Lésion exceptionnelle: genou flottant bilateral

**DOI:** 10.11604/pamj.2014.19.28.4974

**Published:** 2014-09-12

**Authors:** Younes Ouchrif, Issam Elouakili

**Affiliations:** 1 Service de Chirurgie Orthopédique, CHU de Rabat, Maroc

**Keywords:** Genou flottant, lésion, traumatisme des deux membres, floating knee, lesion, trauma of the two limbs

## Image en medicine

Le genou flottant est une entité lésionnelle décrite pour la première fois en 1975 par Blake et McBride. L'atteinte bilatérale est exceptionnelle. Elle survient pour des traumatismes à haute énergie et est généralement accompagnée d'autres lésions potentiellement vitales. Notre patient a présenté à la suite d'une chute d'une hauteur élevée un traumatisme des deux membres inférieurs. Le diagnostic évoqué cliniquement a été confirmé après bilan radiologique standard. La classification de Fraser permet de distinguer 4 types de genou flottant en fonction du siège des fractures. Le traitement est chirurgical, plusieurs moyens d'ostéosynthèses sont possibles. Chez notre patient nous avant optés vu le siège des fractures pour un enclouage des fémurs (rétrograde) et des tibias. Les complications sont très fréquentes, d'abord d'ordre générales qui peuvent causer le décès du patient (embolies graisseuse, embolies pulmonaires, lésions associées) et locales (infection, cal vicieux, pseudarthroses, raideurs des genoux).

**Figure 1:**
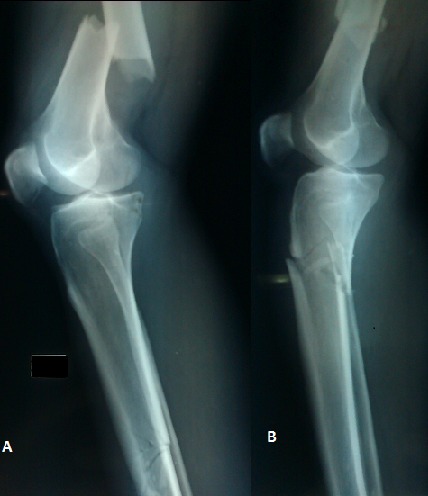
A) incidence de profil montrant le genou flottant droit; B) incidence de profil montrant le genou flottant gauche

